# Do polyphenols affect body fat and/or glucose metabolism?

**DOI:** 10.3389/fnut.2024.1376508

**Published:** 2024-06-11

**Authors:** Saleha Alqarni, Mashael Alsebai, Batool Adal Alsaigh, Abeer Sayer Alrashedy, Israa Talal Albahrani, Albandri Yousef Aljohar, Amjad Obaid Alazmi

**Affiliations:** ^1^Department of Clinical Nutrition, College of Applied Medical Science, King Faisal University, Al-Ahsa, Saudi Arabia; ^2^Department of Clinical Nutrition, Nottingham University, Nottingham, United Kingdom

**Keywords:** polyphenols, antioxidants, type 2 diabetes, obesity, lipid

## Abstract

**Background:**

Obesity is reaching epidemic proportions with 51% of the population expected to be obese by 2030. Recently, polyphenols have been highlighted as an effective approach to managing obesity and associated risks. Polyphenols are a large class of bioactive plant compounds classified into two major categories: flavonoids which are distinguished by the fundamental C6-C3-C6 skeleton and non-flavonoids.

**Objective:**

This systematic review evaluated the effect of different polyphenol sources in overweight and obese people with and without type 2 diabetes. The primary outcome was lipid profile and the secondary outcomes were blood glucose, HbA1c (%), HOMA-IR, weight, and body mass index.

**Method:**

A search was undertaken in PubMed, Web of Science, Medline, and Wiley for randomized control trials that assessed different sources of polyphenols in overweight and obese people with or without type 2 diabetes. The quality of the included studies was assessed using the National Heart, Lung, and Blood Institute Quality Assessment Tool.

**Result:**

The search yielded 935 studies, of which six randomized control trials met the inclusion criteria. Five studies found no significant difference in lipid profile between the control and intervention groups in triglycerides, total cholesterol, LDL cholesterol, and HDL cholesterol. However, one study showed significant differences in triglycerides (*p* = 0.04) and HDL cholesterol (*p* = 0.05) between the two groups with no significant difference in total cholesterol and LDL cholesterol. There were no significant changes in blood glucose observed in the included studies, with only two studies reporting a significant difference in A1c between the groups. Four studies found no difference in HOMA-IR, while one study showed a significant decrease in HOMA-IR in the intervention group compared to the control group. Three studies reported no difference in BMI or weight between the two groups.

**Conclusion:**

The data associated with the specific health benefits of polyphenols and their sources in people with overweight, obese, and type 2 diabetes are still limited, so further research is required to support their use and prove their benefits.

## Introduction

Obesity has reached epidemic proportions, with the World Health Organization (WHO) estimating that there are more than 1.9 billion overweight adults worldwide, of which 650 million are obese (1). In addition, there are more than 340 million overweight or obese children and adolescents between the ages of 5 and 19 (1). By 2030, 51% of the population is expected to be obese (2). Excess weight is associated with a range of metabolic complications such as hypertension, insulin resistance, dyslipidaemia, and type 2 diabetes (3–5), as well as a significant economic burden. The direct costs include healthcare costs for treating related diseases, while indirect costs include lost productivity due to disability or premature death (2, 6). Altering lifestyles to reduce calorie consumption and increase physical activity are difficult to preserve in the long term, therefore, it is necessary to find methods to lower the prevalence of obesity and its associated diseases.

Recently, polyphenols have been highlighted as a practical approach to managing obesity and associated risks. Polyphenols are an enormous class of bioactive plant compounds classified into two major categories: flavonoids which are distinguished by the fundamental C6-C3-C6 skeleton, and non-flavonoids (particularly phenolic acids, stilbenes, and lignans) (7) as shown in Table 1. Flavones, flavonols, isoflavones, flavanones, anthocyanins, and flavanols, commonly known as catechins, are the subclasses that result from the heterocyclic ring connecting the two aromatic rings (7), the primary forms are either conjugated with acid-alcohol or with glycosides in plant food items (7, 8). In addition, polyphenols exist as monomers and oligomers, which are typically referred to as tannins (8). Condensed tannins and hydrolysable tannins are derived from catechin and are usually called procyanidins (8). Phenolic compounds are challenging to quantify because of their diversity and complexity (7, 8). As Polyphenols represent a diverse group of phytochemicals found abundantly in plant-based foods as shown in Table 1.

**Table 1:** Classes of polyphenols and the principal dietary constituents.

Class	Sub-class	Dietary sources
Flavonoids	Flavones	Plants and spices like thyme, rosemary, oregano, and parsley Fruits: celery and olives Vegetables: hot peppers and celery hearts
	Isoflavones	Grape seed/skin Soybean, soy nuts, soy flour/bread, tofu, miso, soy milk, and tofu yoghurt are among the products made from legumes
	Anthocyanidins	Elderberries, strawberries, cherries, plums, cranberries, blackberries, black currants, blueberries, black grapes, blackberries, blackberries, and pomegranate juice
	Flavanones	Lemon, lime orange, orange, grapefruit, tangerine, peppermint
	Flavanols	Apples, apricots, grapes, peaches, nectarines, pears, plums, raisins, raspberries, cherries, blackberries, blues, and cranberries White wine, black tea, dark chocolate, green tea, and cacao
Non-flavonoids	Stilbenes	Grapes, rhubarb and peanuts
	Lignans	Nuts, seeds, rye, wheat, oats, barley, soybean, apricots and strawberries, broccoli, cabbage
	Hydroxybenzoic acids	Strawberries, raspberries, grape juice (black/green) pomegranate juice
	Hydroxycinnamic acids	Tea, coffee, spinach, potato, lettuce, orange, grapefruit, cherry juice, apple juice, lemon, peach, cranberry, pear, cherry (sweet), apple, orange, grapefruit, pear juice, and cider

### Flavonoids

This subgroup constitutes the largest and most studied class of polyphenols. Flavonoids are further categorized into subclasses such as flavonols, flavones, flavanones, flavan-3-ols (including catechins), anthocyanins, and isoflavones. They are found in fruits, vegetables, tea, wine, and various herbal remedies (9, 10).

### Phenolic acids

These are aromatic compounds found in various plant foods, particularly in fruits (e.g., berries), vegetables, whole grains, and beverages like coffee. Major subclasses include hydroxybenzoic acids (e.g., gallic acid) and hydroxycinnamic acids (e.g., caffeic acid, ferulic acid) (9, 11).

### Lignans

Lignans are polyphenolic compounds abundant in seeds, whole grains, fruits, and vegetables. They are converted by intestinal bacteria into enterolignans, which have been associated with various health benefits, including hormone regulation and antioxidant activity (9, 11).

### Stilbenes

This subgroup includes compounds such as resveratrol, primarily found in grapes, red wine, peanuts, and berries. Resveratrol has gained attention for its potential health-promoting effects, including anti-inflammatory and cardioprotective properties (9, 11).

### Others

This category encompasses a wide range of polyphenolic compounds, including tannins, stilbenoids, and curcuminoids. Tannins are commonly found in tea, wine, and certain fruits, and they contribute to the astringent taste of these foods. Stilbenoids, besides resveratrol, include pterostilbene, found in blueberries and grapes (9, 11). Curcuminoids are derived from turmeric and exhibit various biological activities, including antioxidant and anti-inflammatory properties.

**Figure 1:**
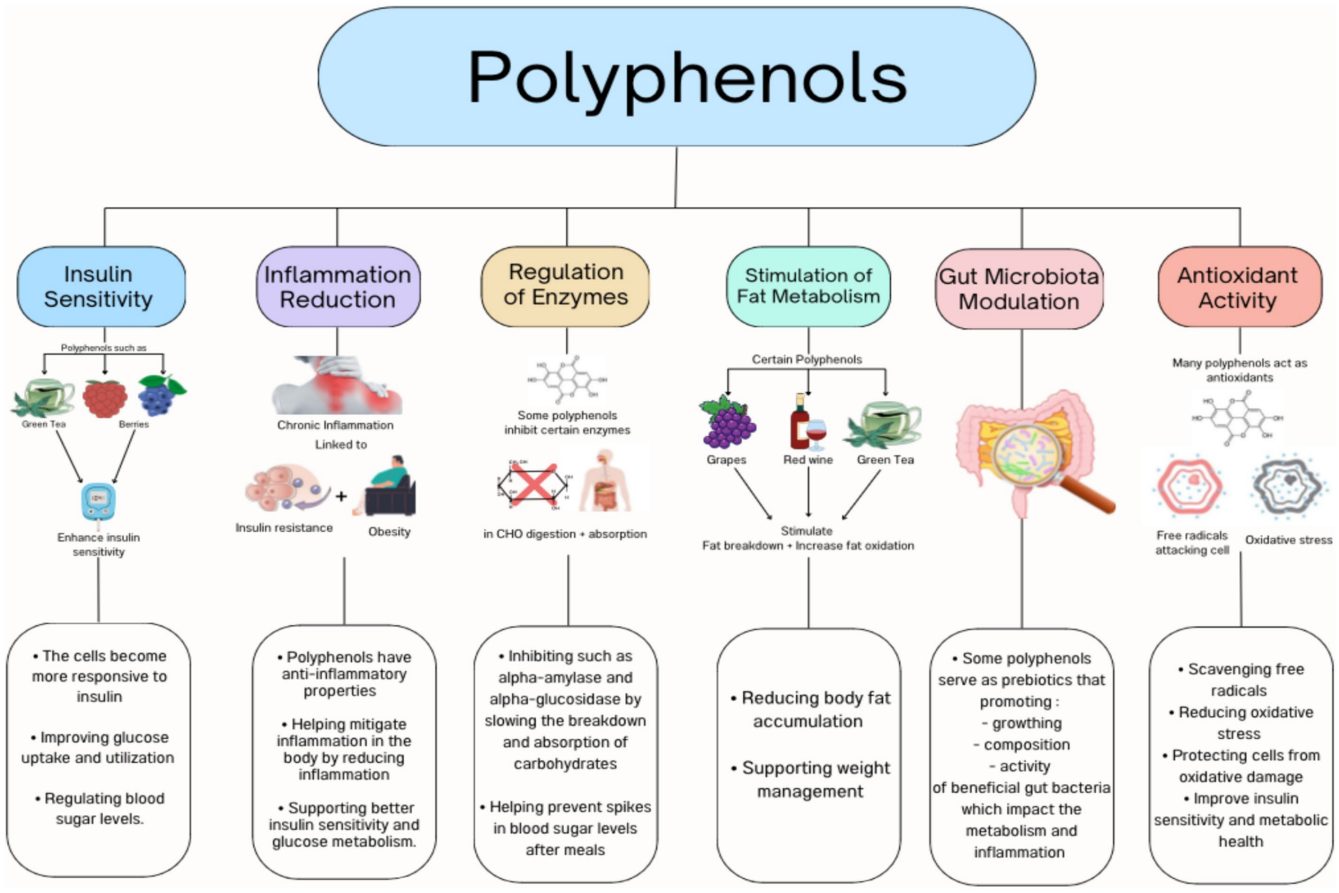
Polyphenols mechanisms.

There are several possible mechanisms for the positive effect of polyphenols in obesity and associated risks as shown in Figure 1 including the inhibition of fat absorption from the gastrointestinal tract, glucose uptake by skeletal muscles, the regulation of fat production, appetite suppression, the modulation of the gut microbiota, and reduced chronic inflammation associated with obesity (12, 13).

Numerous epidemiological and clinical studies have investigated the effect of different polyphenols on obesity and associated risks. For example, Shrime et al. (14), revealed that consuming cocoa, which is high in flavanol, reduced insulin resistance. Also, insulin resistance was improved in healthy individuals who consumed pure-epicatechin for a month by alterations in fasting insulin levels without affecting fasting glucose levels (15). In a systematic analysis of six studies, the green coffee extract reduced fasting blood glucose, while only larger dosages helped enhance insulin resistance (16). Following a 12-week olive leaf polyphenols supplementation in middle-aged, overweight men had increased insulin action and secretion (17). A systematic review and meta-analysis of six studies reported that pistachio nut consumption enhanced insulin resistance (18). Pecan and almond eating appear to improve insulin resistance (19, 20). However, a systematic review of walnuts found no impact on fasting blood glucose or other measures of insulin resistance (21).

Habitual consumption of tea was linked to lower body fat in a cross-sectional human study in Taiwan (22). In addition, a longitudinal investigation from the Netherlands showed a lower body mass index among women with higher dietary flavone, flavonol, and catechin consumption (23). Furthermore, clinical trials have shown a positive effect of high doses of catechins in tea drinks, effectively reducing body fat and body weight, especially when combined with an exercise regimen (24, 25). Moreover, a recent meta-analysis showed that consuming resveratrol significantly reduces weight, body mass index, and fat mass (26). However, a systematic review and meta-analysis of 19 RCTs focusing on the effect of anthocyanin on cardiometabolic biomarkers showed that there is no significant effect of anthocyanin supplementation on body mass index, body weight, waist circumference, and blood pressure (27).

Inconsistencies in the findings and methodological limitations of individual studies, as well as inconsistencies in the results of previous systematic reviews and meta-analyses, justify the need for a comprehensive systematic review to assess the impact of polyphenols on obesity and its associated risks. In contrast to previous systematic reviews, different types of polyphenols were included and compared. In addition to the increasing volume of new studies, an updated systematic review is needed. Therefore, this systematic review compared different sources of polyphenols in overweight and obese individuals with or without type 2 diabetes. The primary outcome was lipid profile and the secondary outcomes were blood glucose, HbA1c (%), HOMA-IR, weight, and body mass index.

## Methodology

This systematic review was conducted according to The Preferred Reporting Items for Systematic Reviews and Meta-Analyses (PRISMA) checklist (28).

### Search strategy

A systematic search of the PubMed, Web of Science, Medline and Wiley databases was performed from 13th May 2023 to Jane 2023 using the following terms: “(“resveratrol (3,5,4’trihydroxystilbene)” OR “epigallocatechin 3 gallate” OR “epigallocatechin gallate (ECG)” OR “epigallocatechin” OR “quercetin” OR “flavonoids” OR “Polyphenols” OR “tannins” OR “lignans” OR “stilbenes” OR “green tea catechins” OR “catechins” OR “epicatechin” OR “procyanidin” OR “genistein” OR “provinols” OR “phenolic acids” OR “apigenin” OR “blackberry polyphenols” OR “curcumin” OR “grape polyphenols” OR “cranberry polyphenols” OR “strawberry polyphenols”) AND (“insulin resistant” OR “insulin receptor substrate” OR “insulin receptor pathway” OR “GLUT” OR“insulin sensitivity”) AND (“Lipid” OR “HDL” OR “LDL” OR “Total cholesterol” OR “Triglycerides”) AND (“fat accumulation” OR “obesity” OR “overweight” OR “hyperlipidemia” AND “glucose” OR “blood sugar”) AND (“clinical trial”). In addition to manual search in references list. All identified studies were saved in EndNote and duplicates were removed.

The criterion for selecting the types of foods presented in the search terms was based on their known richness in polyphenols, as documented in scientific literature. We considered foods that are commonly recognized for their high polyphenol content and relevance to human consumption. Additionally, we aimed to include a diverse range of food sources to provide a comprehensive overview of polyphenol intake. However, there are many other sources of polyphenol not included in our search terms, including olives oil and that should be considered in future research.

### Eligibility criteria

The current systematic review examined study eligibility using a population comparison and outcome as shown in Table 2:

- Study design: randomized control trial to address the research question
- Population: Overweight and obese adults with or without type 2 diabetes
- Intervention: different sources of polyphenols
- Outcomes: The primary outcome is lipid profile and secondary outcomes are blood glucose, HbA1c (%), weight and body mass index.

**Table 2:** Population–intervention–comparison–outcome (PICOS) criteria for study inclusion and exclusion.

Criteria category	Inclusion	Exclusion
Population	Overweight and obese adults with or without type 2 diabetes	Normal weight adults Children/animals
Intervention	Polyphenols	The intervention is not polyphenols
Comparators	Overweight and obese adults with or without type 2 diabetes not treated with polyphenols	Normal weight adults Children/animals
Outcome measure	Primary outcome is lipid profile and secondary outcomes are blood glucose, HbA1c (%), HOMA-IR, weight and body mass index	Lipid profile not assessed
Study design	Randomised control trials	Observational studies, review studies, case reports, pilot studies

### Exclusion criteria

- Review studies
- Case reports
- Observation study
- Non-English studies
- The full text of the study was not available.

### Data extraction and synthesis

The following data were extracted from the studies: the author’s name, the year of publication, the study location, the study design, the target population, the sample size, the baseline characteristics of participants, and the primary outcome lipid profile. The secondary outcomes were blood glucose, weight, and body mass index. A meta-analysis was not performed due to the heterogeneity of the data.

### Quality assessment

The study quality was evaluated using the National Heart, Lung, and Blood Institute (NHLBI) Quality Assessment Tool for randomized controlled trials (29). The tool comprises 14 questions answered using the following options: yes, no, not reported, or not applicable.

## Results

### Eligibility of studies

**Figure 2:**
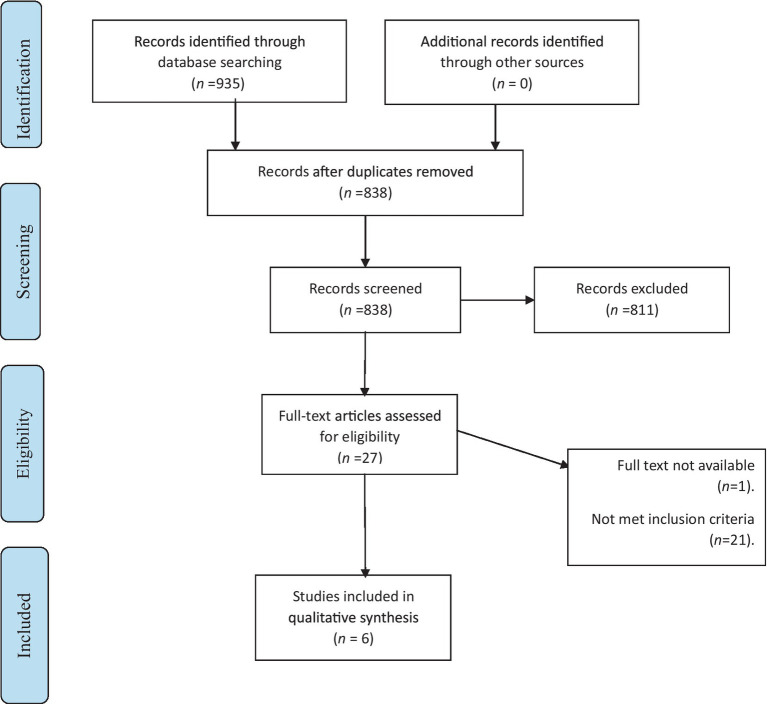
Stages of selecting studies.

The literature search retrieved 935 studies, of which 97 were duplicate studies. Further screening of the title and abstract led to the exclusion of 811 studies, leaving 27 studies to be assessed against the inclusion/exclusion criteria and finally, six studies were included in this review (Figure 2).

### Quality assessment

The quality of each study was evaluated using the NHLBI Quality Assessment Tool for randomized controlled trials (Table 3). Overall, the studies reported on the effect of different sources of polyphenols in overweight and obese people with and without type 2 diabetes seem convergent and reliable to indicate the effect of polyphenols.

**Table 3:** NHLBI tool for quality assessment of included studies.

	Criteria	Kirch et al. (30)	Paquette et al. (31)	Stendell-Hollis et al. (32)	Woerdeman et al. (33)	Aghasi et al. (34)	Asadi et al. (35)
1.	Was the study described as randomised, a randomised trial, a randomised clinical trial, or an RCT?	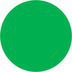	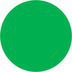	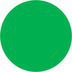	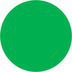	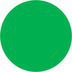	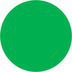
2.	Was the method of randomization adequate (i.e., use of randomly generated assignment)?	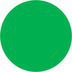	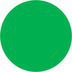	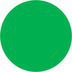	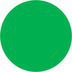	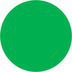	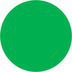
3.	Was the treatment allocation concealed (so that assignments could not be predicted)?	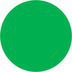	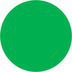	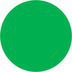	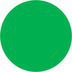	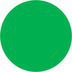	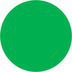
4.	Were study participants and providers blinded to treatment group assignment?	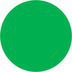	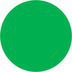	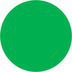	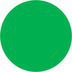	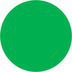	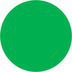
5.	Were the people assessing the outcomes blinded to the participants’ group assignments?	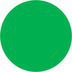	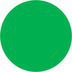	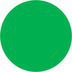	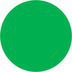	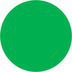	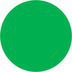
6.	Were the groups similar at baseline on important characteristics that could affect outcomes (e.g., demographics, risk factors, co-morbid conditions)?	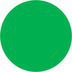	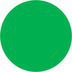	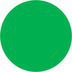	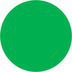	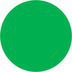	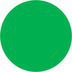
7.	Was the overall drop-out rate from the study at endpoint 20% or lower of the number allocated to treatment?	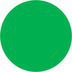	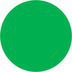	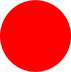	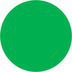	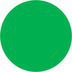	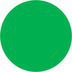
8.	Was the differential drop-out rate (between treatment groups) at endpoint 15 percentage points or lower?	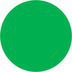	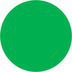	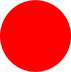	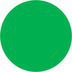	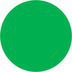	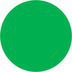
9.	9. Was there high adherence to the intervention protocols for each treatment group?	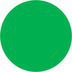	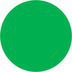	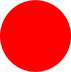	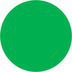	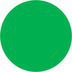	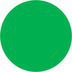
10.	Were other interventions avoided or similar in the groups (e.g., similar background treatments)?	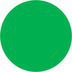	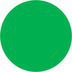	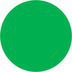	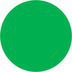	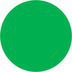	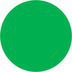
11.	Were outcomes assessed using valid and reliable measures, implemented consistently across all study participants?	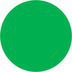	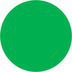	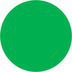	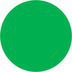	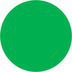	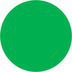
12.	Did the authors report that the sample size was sufficiently large to be able to detect a difference in the main outcome between groups with at least 80% power?	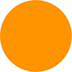	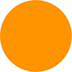	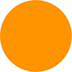	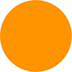	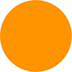	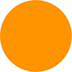
13.	Were outcomes reported or subgroups analyzed prespecified (i.e., identified before analyses were conducted)?	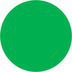	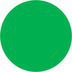	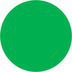	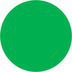	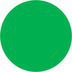	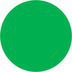
14.	Were all randomszed participants analysed in the group to which they were originally assigned, i.e., did they use an intention-to-treat analysis?	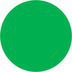	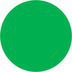	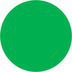	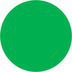	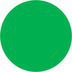	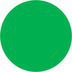

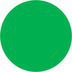
+, yes; 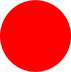 − , no; 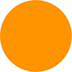
?, cannot determine; 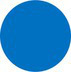
a, not applicable; 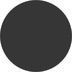
r, not reported.

### Study characteristics

The six included studies, which involved a total of 301 adults, were published between 2010 and 2019. Four studies were conducted on people who were obese or overweight (30–33), and two studies were conducted on people with type 2 diabetes (34, 35).

Different sources of polyphenols were assessed including epicatechin (30), strawberry and cranberry (31), green tea (32), red wine (33), green cardamom (34), and *Melissa officinalis* (35). The study characteristics and results are shown in Tables 4, 5.

**Table 4:** Obesity and polyphenols.

Obesity and polyphenols						
Authors, year	Study design	Location	Interventions vs. control	Baseline characteristics of the participants	Post treatment characteristics of the participants	Results *p*-value
Kirch et al. (30)	RCT	Germany	Intervention (*n*) = 23 25 mg-epicatechin for 2 weeks	Wt = 103.0 ± 3.8 BMI = 32.8 ± 0.8 Triglycerides = 2.22 ± 0.19 Total cholesterol = 5.92 ± 0.14 LDL cholesterol = 3.80 ± 0.12 HDL cholesterol = 1.35 ± 0.06 HOMA-IR = 2.83 (2.00, 4.27) Glucose = 5.72 (5.38, 6.00)	Wt = 102.8 ± 3.8 BMI = 32.7 ± 0.8 Triglycerides = 2.231 ± 0.27 Total cholesterol = 5.73 ± 0.15 LDL cholesterol = 3.76 ± 0.13 HDL cholesterol = 1.28 ± 0.06 HOMA-IR = 2.88 (1.90, 4.39) Glucose = 5.61 (5.22, 6.11)	Daily intake of 25 mg of pure (−)-epicatechin for 2 wk. does not reduce cardiometabolic risk factors in overweight-to-obese adults (*p* > 0.5). There were no significant changes in the parameters of glucose metabolism (glucose, HOMA-IR).
			Control (*n*) = 24 Placebo for 2 weeks	Wt = 103.2 ± 3.8 BMI = 32.8 ± 0.8 Triglycerides = 2.11 ± 0.17 Total cholesterol = 5.94 ± 0.14 LDL cholesterol = 3.86 ± 0.11 HDL cholesterol = 1.33 ± 0.06 HOMA-IR = 2.86 (1.93, 4.15) Glucose = 5.66 (5.33, 6.11)	Wt = 102.9 ± 3.7 BMI = 32.8 ± 0.8 Triglycerides = 2.24 ± 0.19 Total cholesterol = 5.71 ± 0.16 LDL cholesterol = 3.68 ± 0.12 HDL cholesterol = 1.30 ± 0.06 HOMA-IR = 2.94 (1.82, 4.08) Glucose = 5.55 (5.27, 6.11)	
Paquette et al. (31)	RCT	Canada	Intervention (*n*) = 20 333 mg strawberry and cranberry polyphenols beverage /day for 6 weeks	Wt = 85 ± 3 BMI = 31 ± 1 Triglycerides = 2.03 ± 0.24 Total cholesterol = 5.70 ± 0.17 LDL cholesterol = 3.52 ± 0.17 HDL cholesterol = 1.25 ± 0.05	Wt = − BMI = − Triglycerides = 1.82 ± 0.21 Total cholesterol = 5.60 ± 0.19 LDL cholesterol = 3.51 ± 0.17 HDL cholesterol = 1.26 ± 0.06	No differences in changes from baseline (Post v. Pre) for total, LDL- and HDL-cholesterol or TAG were observed within each group or between the two groups
			Control (*n*) = 21 Placebo for 6 weeks	Wt = 85 ± 3 BMI = 31 ± 1 Triglycerides = 1.73 ± 0.26 Total cholesterol = 5.37 ± 0.22 LDL cholesterol = 3.20 ± 0.15 HDL cholesterol = 1.33 ± 0.05	Wt = − BMI = − Triglycerides = 1.56 ± 0.18 Total cholesterol = 5.45 ± 0.20 LDL cholesterol = 3.37 ± 0.17 HDL cholesterol = 1.37 ± 0.06	
Stendell-Hollis et al. (32)	RCT	USA	Intervention (*n*) = 23 240 mL green tea four times daily for 6 months	Wt = 81.9 ± 15.3 BMI = 31.0 ± 4.3 Triglycerides = 1.8 ± 1.0 Total cholesterol = 5.9 ± 1.0 LDL cholesterol = 3.8 ± 0.9 HDL cholesterol = 1.3 ± 0.39 HOMA-IR = 6.9 (7.4) Glucose = 6.5 (1.3)	Wt = 80.7 ± 14.9 BMI = 30.5 ± 4.2 Triglycerides = 1.6 ± 2.1 Total cholesterol = 5.6 ± 1.0 LDL cholesterol = 3.4 ± 1.1 HDL cholesterol = 1.4 ± 0.4 HOMA-IR = 5.8 (3.4) Glucose = 5.6 (1.0)	After 6 months, there were *no significant* changes in weight, BMI, triglycerides, total cholesterol, HDL and LDL cholesterol, glucose and HOMA-IR between the two groups (*p* > 0.5)
			Control (*n*) = 16 Placebo tea for 6 months	Wt = 77.8 ± 9.8 BMI = 28.7 ± 3.8 Triglycerides = 1.7 ± 0.9 Total cholesterol = 6.9 ± 1.3 LDL cholesterol = 4.7 ± 1.1 HDL cholesterol = 1.4 ± 0.4 HOMA-IR = 4.1 (1.6) Glucose = 7.0 (1.2)	Wt = 78.0 ± 9.1 BMI = 28.8 ± 3.7 Triglycerides = 1.5 ± 0.8 Total cholesterol = 6.3 ± 1.6 LDL cholesterol = 3.9 ± 1.3 HDL cholesterol = 1.6 ± 0.4 HOMA-IR = 7.4 (7.4) Glucose = 7.0 (1.0)	
Woerdeman et al. (33)	RCT	Netherlands	Intervention (*n*) = 14 600 mg/day of red wine polyphenols for 8 weeks	Wt = 108 ± 15.2 BMI = 33.5 Triglycerides = 1.2 ± 1.0 Total cholesterol = 4.8 ± 1.2 LDL cholesterol = 2.9 ± 1.1 HDL cholesterol = 1.2 ± 0.4 HOMA-IR = 3.2 (2.0; 4.5) Glucose = 5.3 ± 0.5	Wt = 109.0 ± 15.8 BMI = − Triglycerides = 1.4 ± 1.1 Total cholesterol = 4.8 ± 1.1 LDL cholesterol = 3.0 ± 1.1 HDL cholesterol = 1.2 ± 0.4 HOMA-IR = 2.9 (2.1; 3.8) Glucose =5.3 ± 0.7	There were no significant changes in either total, LDL or HDL cholesterol or triglyceride levels, glucose, or HOMA-IR after red wine supplementation between group			
			Control (*n*) = 15 Placebo for 8 weeks	Wt = 106.4 ± 16.1 BMI = 33.1 Triglycerides = 1.1 ± 0.7 Total cholesterol = 4.5 ± 1.1 LDL cholesterol = 2.8 ± 1.0 HDL cholesterol = 1.2 ± 0.3 HOMA-IR = 2.3 (1.4; 2.7) Glucose = 5.0 ± 0.7	Wt = 106.6 ± 16.8 BMI = – Triglycerides = 1.0 ± 0.8 Total cholesterol = 4.4 ± 1.0 LDL cholesterol = 2.7 ± 1.0 HDL cholesterol = 1.3 ± 0.3 HOMA-IR = 2.2 (1.5; 2.8) Glucose = 5.2 ± 0.5		

Values are presented as mean ± standard deviation. Wt = weight; BMI = body mass index; HOMA-IR, homeostasis model assessment-insulin resistance; HDL, high-density lipoprotein; LDL, low-density lipoprotein; (−) not available.

**Table 5:** Type 2 diabetes mellitus and polyphenol.

Type 2 diabetes mellitus and polyphenol						
Author, years	Study design	Location	Intervention vs. control	Baseline characteristics of the participants	Posttreatment characteristics of the participants	Results *p*-value
Aghasi et al. (34)	RCT	Iran	Intervention (*n* = 41) 3 g of green cardamom supplement for 10 weeks	BMI = 29.06 ± 3.21 HbA1c (%) = 8.19 (0.68) HOMA-IR = 5.01 (1.90) Triglycerides = 158.4 (1.62) Total cholesterol = 155.7 (33.2) LDL cholesterol = 80.2 (20.3) HDL cholesterol = 41.6 (1.2) Glucose = 159.5 (38.7)	BMI = 28.8 ± 3.1 HbA1c (%) = 7.71 (0.67) HOMA-IR = 3.80 (1.65) Triglycerides = 125.8 (1.5) Total cholesterol = 153.43 (34.7) LDL cholesterol = 77.9 (20.4) HDL cholesterol = 41.6 (1.2) Glucose = 146.8 (27.07)	After 10 weeks, There were no significant changes in serum glucose TC, HDL-c and LDL-c levels between the two groups. A significant decrease in serum HbA1C (−0.4%), HOMA-IR (−1.7) and TG (−39.9 mg dL − 1) was observed in the cardamom group
			Control group (*n* = 42). Placebo	BMI = 28.66 ± 4.34 HbA1c (%) = 7.27 ± 0.60 HOMA-IR = 1.50 ± 0.51 Triglycerides = 135.54 ± 51.55 Total cholesterol = 142.48 ± 33.55 LDL cholesterol = 70.53 ± 26.19 HDL cholesterol = 44.83 ± 10.87 Glucose = 143.09 ± 37.39	BMI = 29.2 ± 3.5 HbA1c (%) = 7.72 (0.58) HOMA-IR = 4.46 (1.65) Triglycerides = 138.03 (1.44) Total cholesterol = 160.5 (30.27) LDL cholesterol = 87.4 (22.8) HDL cholesterol = 41.6 (1.23) Glucose = 145.8 (23.2)	
Asadi et al. (35)	RCT	Iran	Melissa. officinalis capsules (700 mg/d; *n* = 31) twice	BMI = 28.66 ± 4.34 HbA1c (%) = 7.27 ± 0.60 HOMA-IR = 1.50 ± 0.51 Triglycerides = 135.54 ± 51.55 Total cholesterol = 142.48 ± 33.55 LDL cholesterol = 70.53 ± 26.19 HDL cholesterol = 44.83 ± 10.87 Glucose = 143.09 ± 37.39	BMI = − HbA1c (%) = 6.99 ± 0.68 HOMA-IR = 1.30 ± 0.52 Triglycerides = 122.03 ± 42.74 Total cholesterol = 145.38 ± 31.15 LDL cholesterol = 72.46 ± 28.85 HDL cholesterol = 48.51 ± 10.55 Glucose = 143.29 ± 33.59	After 12 weeks, there were significant differences in HbA1c (*p* = 0.002), TG (*p* = 0.04), HDL-c (*p* = 0.05) between the two group
			Control group (*n* = 31). Placebo	BMI = 28.37 ± 3.71 HbA1c (%) = 7.36 ± 0.49 HOMA-IR = 1.56 ± 0.62 Triglycerides = 143.19 ± 62.19 Total cholesterol = 147.09 ± 31.85 LDL cholesterol = 70.53 ± 26.19 HDL cholesterol = 44.64 ± 9.23 Glucose = 138.87 ± 34.59	BMI = − HbA1c (%) = 7.47 ± 0.74 HOMA-IR = 1.42 ± 0.67 Triglycerides = 138.67 ± 38.52 Total cholesterol = 153.09 ± 26.97 LDL cholesterol = 70.53 ± 26.19 HDL cholesterol = 44.61 ± 9.29 Glucose = 151.80 ± 35.34	

Values are presented as mean ± standard deviation. Wt = weight; BMI = body mass index; HOMA-IR, homeostasis model assessment-insulin resistance; HDL, high-density lipoprotein; LDL, low-density lipoprotein; (−) not available.

### Primary outcomes

#### Polyphenols and lipid profile

Six studies evaluated the lipid profile, five of which found no significant difference in triglycerides, total cholesterol, LDL cholesterol and HDL cholesterol between the control and the intervention groups (30–34). However, Asadi et al. (35) showed significant differences in TG (*p* = 0.04) and HDL-c (*p* = 0.05) between the two groups at the end of the study.

### Secondary outcomes

#### Polyphenols and glucose level

There were no significant changes in glucose levels between the groups (30–35).

#### Polyphenols and A1c

Only two studies assessed cumulative glucose and found significant differences between groups (34, 35).

#### Polyphenols and HOMA-IR

Five studies evaluated HOMA-IR, four of which found no difference between the two groups (30, 32, 33, 35) while Aghasi et al. (34) showed that there is a significant decrease in HOMA-IR (−1.7) in the intervention group compared to the control group.

#### Polyphenols and BMI

Three studies assessed BMI and found no difference between the two groups (30, 32, 34).

#### Polyphenols and weight

Three studies measured weight and showed that there is no difference between the two groups (30, 32, 33).

## Discussion

This systematic review compared the effects of different sources of polyphenols in overweight and obese people with or without type 2 diabetes. The primary outcome was lipid profile and the secondary outcomes were blood glucose, HbA1c (%), HOMA-IR, weight, and body mass index. However, the effects of different sources of polyphenols for obese or overweight adults with or without type 2 diabetes remain unclear.

### Epicatechin

Epicatechin is a polyphenol found in various plant-based foods and beverages such as cocoa, grapes, apples, berries, hazelnuts, walnuts, and tea. Kirch et al. (30) investigated whether regular consumption of 25 mg of pure epicatechin can affect glucose, HOMA-IR, LDL, HDL, total cholesterol, and triglycerides in overweight or obese people, showing that 2 weeks of epicatechin supplementation did not reduce cardiometabolic risk factors with no significant changes in glucose metabolism (glucose, HOMA-IR). Similarly, both Dower et al. (15) and Gutiérrez-Salmeán et al. (36) showed that regular intake of pure epicatechin (25–100 mg/day) was not effective or less effective on cardiovascular disease risk factors than comparative cocoa intake. Meta-analyses of randomized controlled trials found positive effects of cocoa consumption on glucose and lipid metabolism (14, 37) that could be predicted by epicatechin intake from cocoa (38). However, the results are inconsistent due to different dosing duration and may be due to adherence.

### Strawberry and cranberry

There are several different types of phenolic compounds in strawberries and cranberries including phenolic acids, flavonoids, and polymerized molecules (39). Several vitro and animal studies have suggested that polyphenols may enhance peripheral glucose absorption in insulin-sensitive areas by enhancing GLUT4 translocation and activity and lowering oxidative stress and inflammation (40, 41). For example, Paquette et al. (31) evaluated the effectiveness of berries and strawberries on insulin sensitivity and lipid profile in overweight, obese, and non-diabetic subjects showing that a six-week intervention with 333 mg of polyphenols from strawberries and cranberries improved insulin sensitivity but was ineffective in improving cardiac risk factors. However, other studies have shown that eating freeze-dried strawberry powder or cranberry extract has a positive influence on lowering total cholesterol and LDL cholesterol (42, 43). This discrepancy may be due to the delivery of strawberries and cranberries in different forms (juice, dried strawberries, and powder) and the different quantities consumed, which makes it difficult to determine their effect.

### Green tea

Epidemiologic and animal research supports the positive effect of regular green tea consumption in decreasing the risk of cardiovascular disease (44) and obesity (45). There are several hypothesized methods by which green tea and its naturally occurring polyphenolic catechins may modify body weight. For instance, green tea and its derivatives cause glucose malabsorption and downregulate fatty acid synthase (46, 47). Individuals with visceral obesity who consumed 583 mg of catechins daily for 12 weeks saw significant reductions in body weight and LDL cholesterol compared to the control group (48). However, Stendell-Hollis et al. (32) evaluated the effect of green tea consumption on weight, body mass index, triglycerides, total cholesterol, HDL and LDL cholesterol, glucose, and HOMA-IR in weight gain showing no significant differences between the participants who consumed 960 mL of decaffeinated green tea or placebo daily for 6 months.

### Red wine

It has been reported that a moderate wine intake might inhibit metabolic syndrome and related medical consequences (49). According to preclinical research, red wine polyphenols are beneficial and improve insulin sensitivity in obese animal models. However, the effect of red wine on humans is minimal. Only one randomized control trial was conducted by Woerdeman et al. (33) to assess the effection of red wine consumption on total cholesterol, HDL and LDL cholesterol, glucose, and HOMA-IR. The participants were randomized to 600 mg/day of red wine polyphenols or placebo daily for 8 weeks and there were no significant changes in either total, LDL or HDL cholesterol or triglyceride levels, glucose, and HOMA-IR after red wine supplementation between groups (*p* > 0.5).

### Green cardamom

Green cardamom is a good source of polyphenols with antioxidant properties (50). Cardamom supplementation enhanced insulin sensitivity and reduced total cholesterol and LDL-cholesterol in prediabetic women (51). However, these outcomes did not differ significantly from the placebo group. According to Azimi et al. (52), the lipid profiles of type 2 diabetes people who consumed three glasses of black tea along with 3 g of cardamom were improved when compared to the control group which only consumed three glasses of black tea. Furthermore, it has been shown that 3 g of cardamom significantly decreased serum HbA1C (−0.4%), HOMA-IR (−1.7), and TG (−39.9 mg dL − 1) in the cardamom group compared to placebo (34). However, there were no significant changes in serum glucose TC, HDL-c, and LDL-c levels between the two groups after 10 weeks (34).

### *Melissa officinalis* (also known as lemon balm)

*Melissa officinalis* is a plant that can lower blood sugar and fat levels due to its abundance of flavonoids (53). Studies have shown the lipid-lowering and anti-inflammatory properties of *M. officinalis* (53, 54). Furthermore, Asadi et al. (35) assessed the effect of *M. officinalis* intake (700 mg/d) versus placebo on HbA1c, triglycerides, total cholesterol, HDL and LDL cholesterol, glucose, and HOMA-IR in individuals with type 2 diabetes, observing significant differences in HbA1c (*p* = 0.002), triglycerides (*p* = 0.04), and HDL-c (*p* = 0.05) between the two groups after 12 weeks. *In vitro* and animal experiments also demonstrated that *M. officinalis* reduced blood glucose and lipids (55, 56).

### Potential mechanisms

Polyphenols are subject to a series of enzymatic reactions and microbial transformations upon ingestion, culminating in their absorption, distribution, metabolism, and excretion throughout various tissues and organs.

First, the chemical structure of polyphenolic compounds greatly influences their metabolic fate. For example, flavonoids, phenolic acids, and other subclasses exhibit distinct metabolic pathways, leading to the formation of diverse metabolites with different bioactivities (57). Phase I and II metabolic reactions, which are mainly facilitated by hepatic enzymes, modify the structure of the polyphenols, making them more water-soluble and leading to their elimination (58, 59).

Moreover, consuming food components together can significantly affect polyphenol metabolism. Interactions with macronutrients, such as lipids and proteins, may affect the absorption kinetics and bioavailability of polyphenols (60). In addition, the presence of other bioactive compounds within food matrices can modify polyphenol metabolism, which may enhance or inhibit their bioactivity through synergistic or antagonistic effects (61).

Furthermore, individual differences in the composition of the gut microbiota contribute to interindividual differences in polyphenol metabolism. Some microbial species possess the enzymatic machinery necessary for the degradation and biotransformation of polyphenolic compounds, resulting in metabolites that may exhibit distinct physiological effects compared to their parent compounds (62).

Understanding the metabolism of polyphenolic compounds is crucial to elucidating their health effects and therapeutic potential. By revealing the complex interplay between nutritional factors, host physiology, and gut microbiota, future research endeavors can pave the way for personalized nutritional interventions aimed at improving the bioavailability of polyphenols and harnessing their beneficial properties for human health and well-being.

## Strength and limitation

The quality assessment of the included studies was conducted to evaluate the methodological rigor and reliability of the evidence presented. We utilized the NHLBI Quality Assessment for Randomized Control Trials (RCTs) which assesses various aspects of study design, conduct, and reporting.

Overall, the quality of the included polyphenol studies varied. Several studies demonstrated a high level of methodological rigor, including randomized controlled trials (RCTs) with appropriate randomization and blinding procedures. However, certain limitations were observed across the studies. Common issues included small sample sizes, lack of blinding, incomplete outcome data, and potential biases.

Despite these limitations, many studies provided valuable insights into the potential health effects of polyphenols.

It is important to interpret the findings of polyphenol studies with caution, considering the methodological limitations identified. Future research should prioritize high-quality study designs, including well-powered RCTs with long-term follow-up, and systematic reviews with rigorous methodology.

## Conclusion

The current systematic review discussed the effect of different sources of polyphenols in overweight and obese people with or without type 2 diabetes, showing that cardamom significantly decreased serum HbA1C, HOMA-IR, and triglyceride and *M. officinalis* reduced blood sugar fat levels and lipids. However, the data associated with the specific health benefits of polyphenols and their sources in people who are overweight, obese, or have type 2 diabetes are still unclear and further research is required to support their use and prove their benefits.

## Data availability statement

The original contributions presented in the study are included in the article/supplementary material, further inquiries can be directed to the corresponding author.

## Author contributions

SA: Conceptualization, Data curation, Formal analysis, Funding acquisition, Investigation, Methodology, Project administration, Resources, Software, Supervision, Validation, Visualization, Writing – original draft, Writing – review & editing. MA: Methodology, Writing – review & editing. BA: Methodology, Writing – review & editing. AbA: Methodology, Writing – review & editing. IA: Methodology, Writing – review & editing. AlA: Methodology, Writing – review & editing. AmA: Methodology, Writing – review & editing.
